# Supplementary material for: Chromosome-level assembly of the horseshoe crab genome provides insights into its genome evolution
Source: Nat Commun. 2020 May 8;11:2322. doi: 10.1038/s41467-020-16180-1 (PMC7210998; doi:10.1038/s41467-020-16180-1)
Supplement: Supplementary file 1 — Supplementary Information [file 41467_2020_16180_MOESM1_ESM.pdf]

Supplementary Information

for

Chromosome-level assembly of the horseshoe crab genome provides insights into its genome evolution

Shingate et al.

Contents

Supplementary Figures 2

Supplementary Figure 1. K-mer plot of the mangrove HSC genome 2

Supplementary Figure 2. Neutral mutation rate of the mangrove HSC 3

Supplementary Figure 3. Hox gene clusters in the mangrove HSC genome 4

Supplementary Figure 4. Paralogous "*Pde4L* loci" in the mangrove HSC genome 5

Supplementary Figure 5. Paralogous "*NotchL* loci" in the mangrove HSC genome 6

Supplementary Figure 6. Paralogous "*Fas1L* loci" in the mangrove HSC genome 7

Supplementary Figure 7. Paralogous "*Mical3L* loci" in the mangrove HSC genome 8

Supplementary Figure 8. Paralogous "*Mbnl2L* loci" in the mangrove HSC genome 9

Supplementary Figure 9. Paralogous "*Syt10l*/*Syt7l* loci" in the mangrove HSC genome 10

Supplementary Figure 10. Paralogous "*Tbx1L* loci" in the mangrove HSC genome 11

Supplementary Figure 11. Paralogous "*Nphs1l*/*Hmcn1l* loci" in the mangrove HSC genome 12

Supplementary Figure 12. Paralogous "Six gene loci" in the mangrove HSC genome 13

Supplementary Figure 13. Paralogous "*Lphn1La*/*Lphn1Lb* loci" in the mangrove HSC genome 14

Supplementary Tables 15

Supplementary Table 1. Genome size estimation using the k-mer method 15

Supplementary Table 2. Assembly statistics of the PacBio-HiC assembly of the mangrove HSC genome 15

Supplementary Table 3. Lengths of the 16 assembled mangrove HSC chromosomes 16

Supplementary Table 4. Comparison of our mangrove HSC genome statistics with other published chelicerate genomes 17

Supplementary Table 5. Repetitive sequences in the mangrove HSC genome assembly 18

Supplementary Table 6. Major Pfam domain families identified in mangrove HSC proteins 19

Supplementary Table 7. Positively enriched GO terms associated with proteins corresponding to genes in tandem gene clusters 20

Supplementary Table 8. Details of the arthropod species used for estimation of the neutral mutation rate and sources of the datasets 22

Supplementary Figures

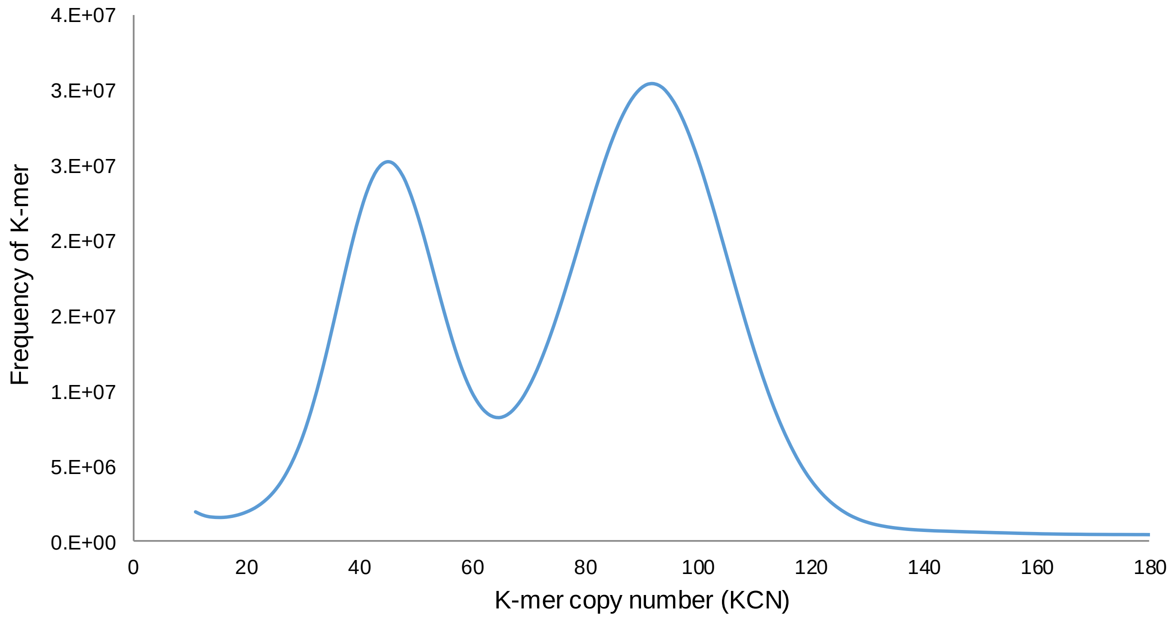

Supplementary Figure 1. K-mer plot of the mangrove HSC genome**.** k-mer plot showing the distribution of k-mer copy number (KCN) at 31-mer for the mangrove HSC genome. For better visualization of KCN distribution, only values between 11 and 180 are shown in the graph. The two peaks reflect the high level of heterozygosity in the genome.The first peak (KCN=46) represents the heterozygous single copy k-mers while the second peak (KCN=92) represents the homozygous single copy k-mers in the genome.

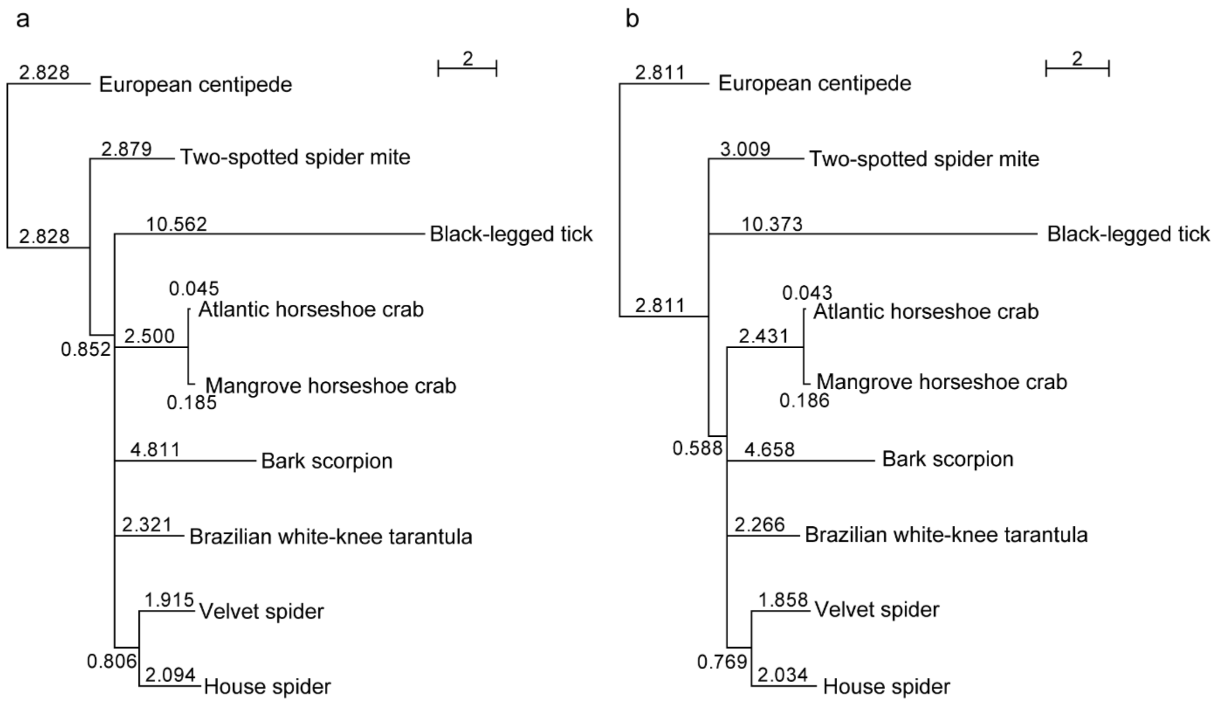

Supplementary Figure 2. Neutral mutation rate of the mangrove HSC. Neutral trees based on four-fold degenerate (4D) sites from 162 one-to-one orthologues are shown. Values above the branches represent the number of substitutions per 4D site for each species. The two trees were generated based on two different topologies – topology obtained from our phylogenomic analysis (a) and the Xiphosura nested within Arachnida topology as shown by a recent study [1] (b).

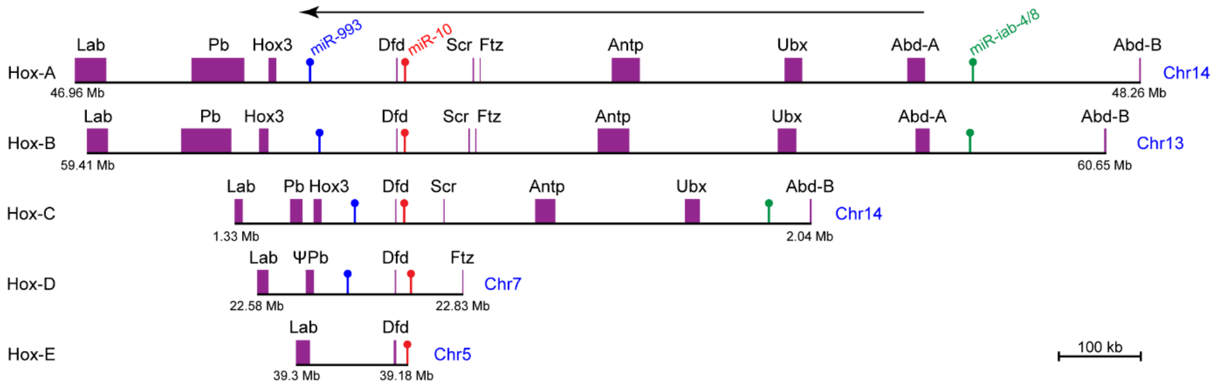

Supplementary Figure 3. Hox gene clusters in the mangrove HSC genome. Pink boxes represent the Hox genes with the black arrow denoting the transcriptional orientation. The relative position of miRNAs are indicated. The start and end position is indicated below each of the clusters.

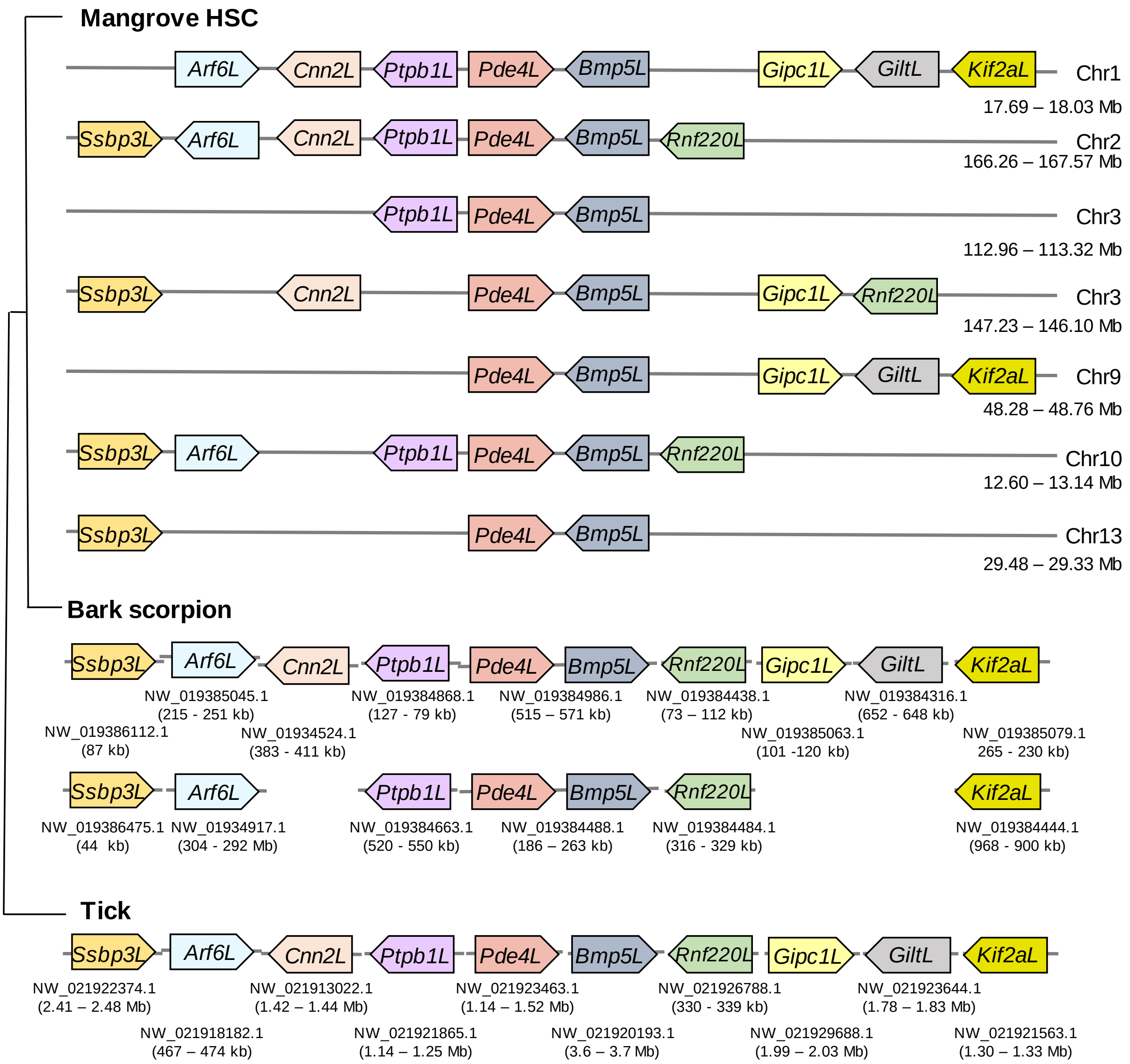

Supplementary Figure 4. Paralogous "*Pde4L* loci" in the mangrove HSC genome. *Pde4L* gene loci from mangrove HSC, bark scorpion and tick are shown. Block arrows denote genes with their directions representing the transcriptional orientation. Paralogous genes are colour-coded. NCBI accession numbers of bark scorpion and tick contigs or scaffolds are shown below the genes. Since some bark scorpion and tick genes are present on separate contigs or scaffolds, their orientations are presumed. *Arf6L*, ADP-ribosylation factor 6-like; *Cnn2L*, calponin 2-like; *Ptbp1L*, polypyrimidine tract-binding protein 1-like; *Pde4L*, cAMP-specific 3',5'-cyclic phosphodiesterase 4-like; *Bmp5L*, bone morphogenetic protein 5-like; *GiltL*, gamma-interferon-inducible lysosomal thiol reductase-like; *Gipc1L*, GIPC PDZ domain containing family member-like; *Kif2AL*, kinesin-like protein KIF2A-like; *Rnf220L*, E3 ubiquitin-protein ligase RNF220-like; *Ssbp3L*,single-stranded DNA-binding protein 3-like.

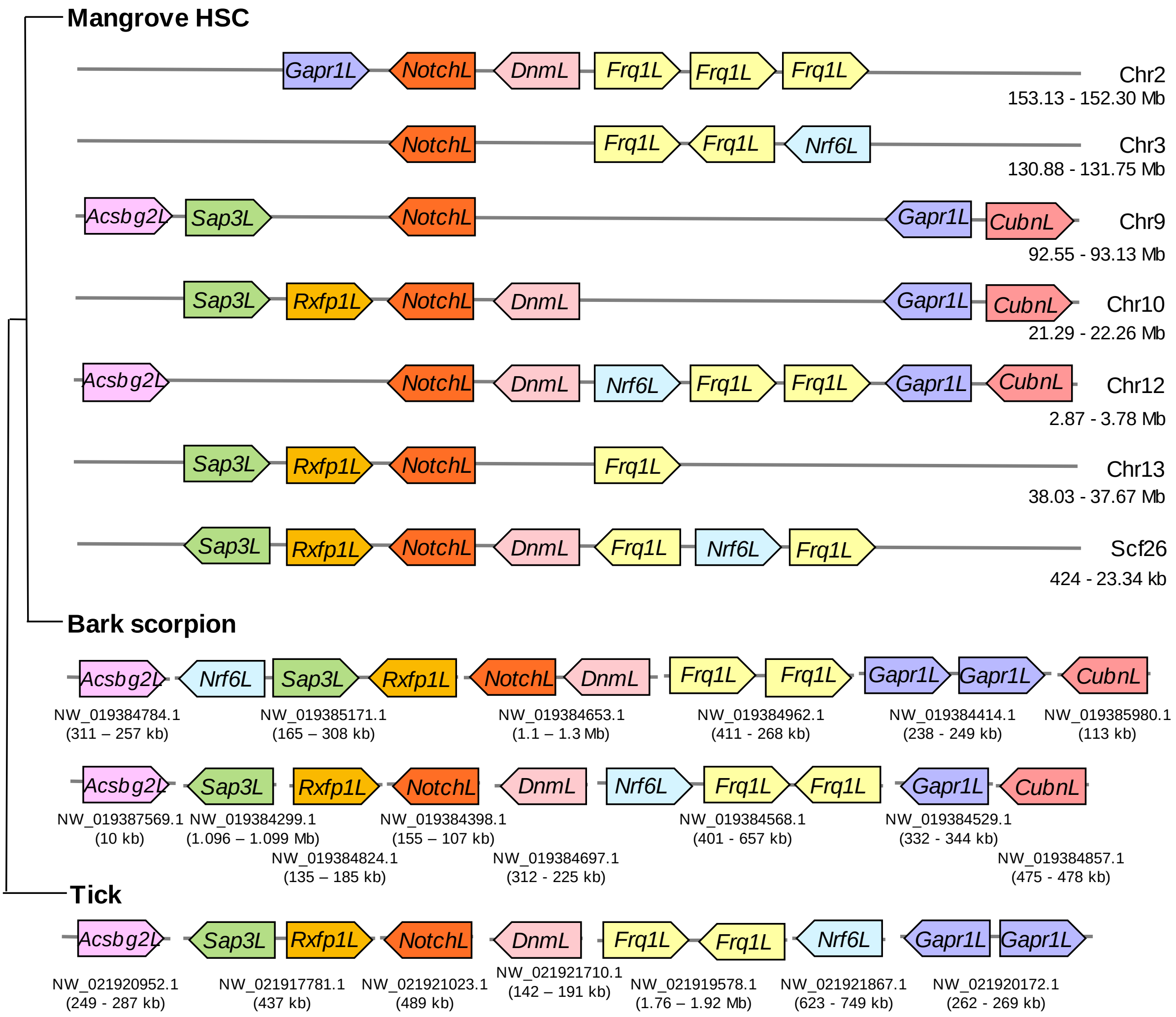

Supplementary Figure 5. Paralogous "*NotchL* loci" in the mangrove HSC genome. *NotchL* gene loci from mangrove HSC, bark scorpion and tick are shown. Block arrows denote genes with their directions representing the transcriptional orientation. Paralogous genes are colour-coded. *Cubnl* is absent in the *Ixodes* genome assembly. NCBI accession numbers of bark scorpion and tick contigs or scaffolds are shown below the genes. Since some bark scorpion and tick genes are present on separate contigs or scaffolds, their orientations are presumed. *NotchL*, neurogenic locus notch protein-like; *DnmL*, dynamin-like; *Frq1L*, frequenin-1-like; *Nrf6L*, nose resistant to fluoxetine protein 6-like; *Rxfp1L*, relaxin receptor 1-like; *Sap-3L*, ganglioside GM2 activator-like; *Gapr1L*, Golgi-associated plant pathogenesis-related protein 1-like: *CubnL*, Cubilin-like; *Acsf2L*, acyl-CoA synthetase family member 2-like.

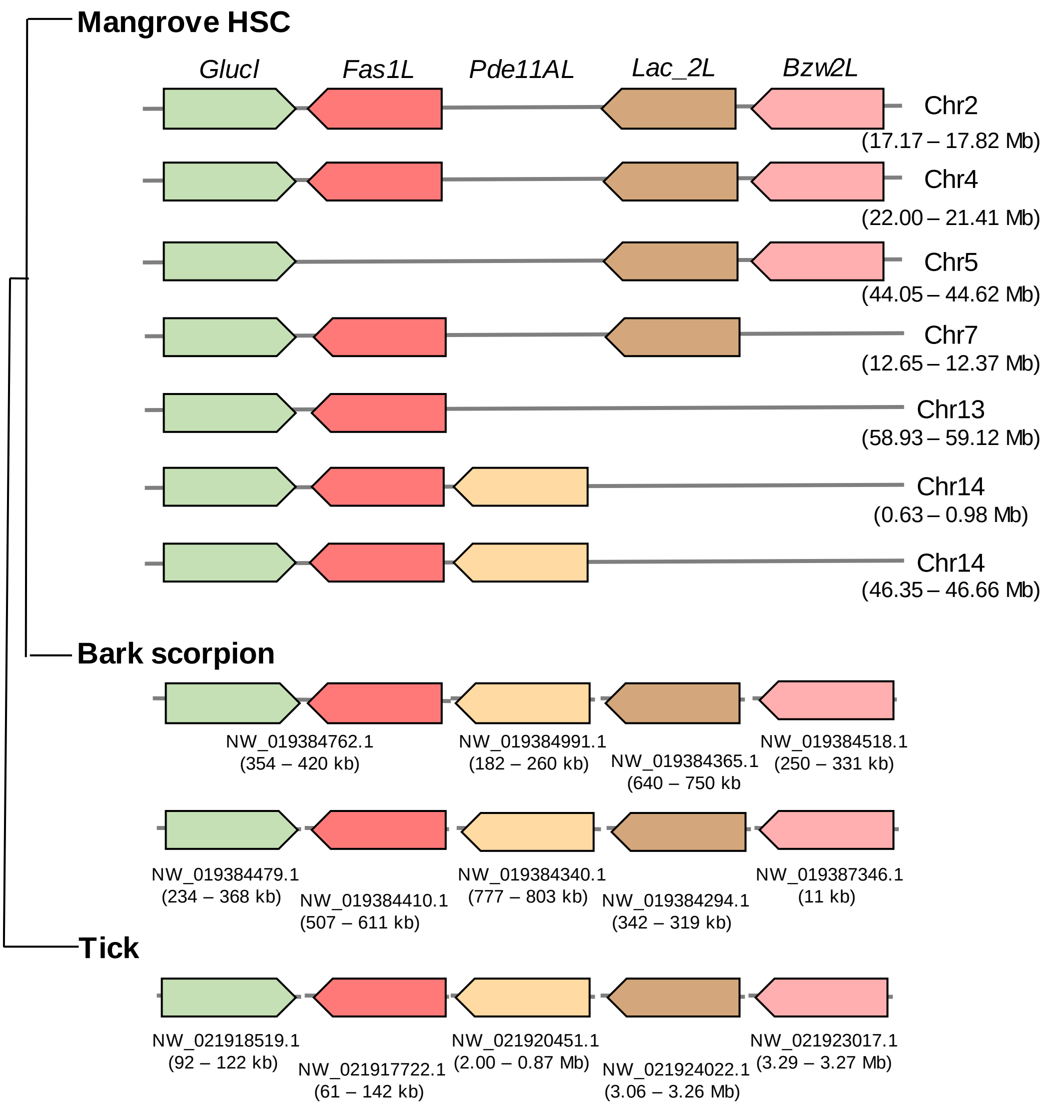

Supplementary Figure 6. Paralogous "*Fas1L* loci" in the mangrove HSC genome. *Fas1L* gene loci from mangrove HSC, bark scorpion and tick are shown. Block arrows denote genes with their directions representing the transcriptional orientation. Paralogous genes are colour-coded. NCBI accession numbers of bark scorpion and tick contigs or scaffolds are shown below the genes. Since some bark scorpion and tick genes are present on separate contigs or scaffolds, their orientations are presumed. *Bzw2L*, basic leucine zipper and W2 domain-containing protein 2-like; *Lac_2L*, lachesin-like; *Fas1L*, fasciclin-1-like; *Glucl*, glutamate-gated chloride channel; *Pde11AL*, dual 3',5'-cyclic-AMP and -GMP phosphodiesterase 11A-like.

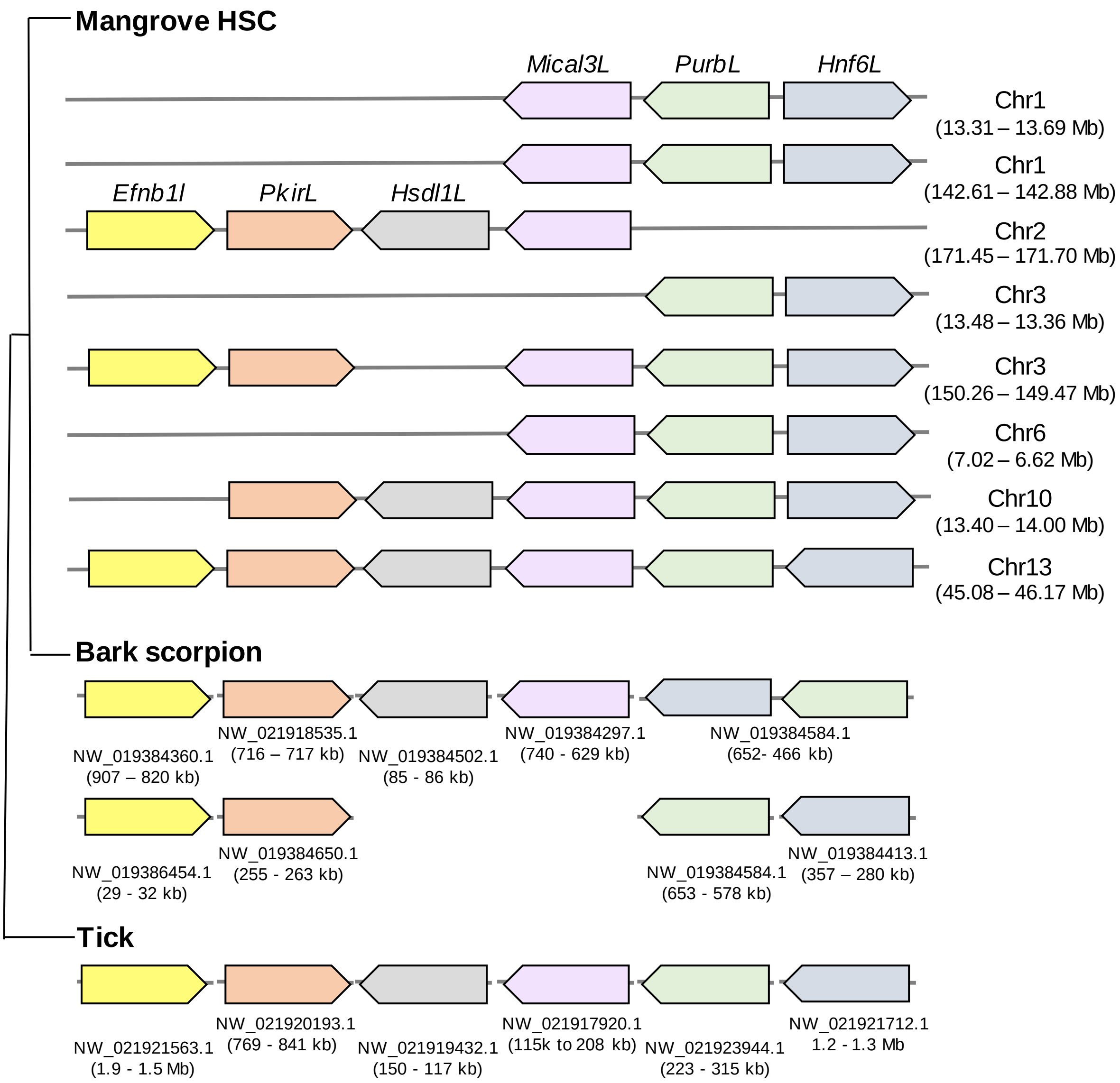

Supplementary Figure 7. Paralogous "*Mical3L* loci" in the mangrove HSC genome. *Mical3L* gene loci from mangrove HSC, bark scorpion and tick are shown. Block arrows denote genes with their directions representing the transcriptional orientation. Paralogous genes are colour-coded. NCBI accession numbers of bark scorpion and tick contigs or scaffolds are shown below the genes. Since some bark scorpion and tick genes are present on separate contigs or scaffolds, their orientations are presumed. *Efnb1L*, ephrin-B1-like; *PkirL*, pyrokinin-1 receptor-like; *Hsdl1*, inactive hydroxysteroid dehydrogenase-like protein 1-like; *Mical3L*, F-actin-methionine sulfoxide oxidase MICAL3-like; *PurbL*, transcriptional activator protein Pur-beta-like; *Hnf6L*, hepatocyte nuclear factor 6-like.

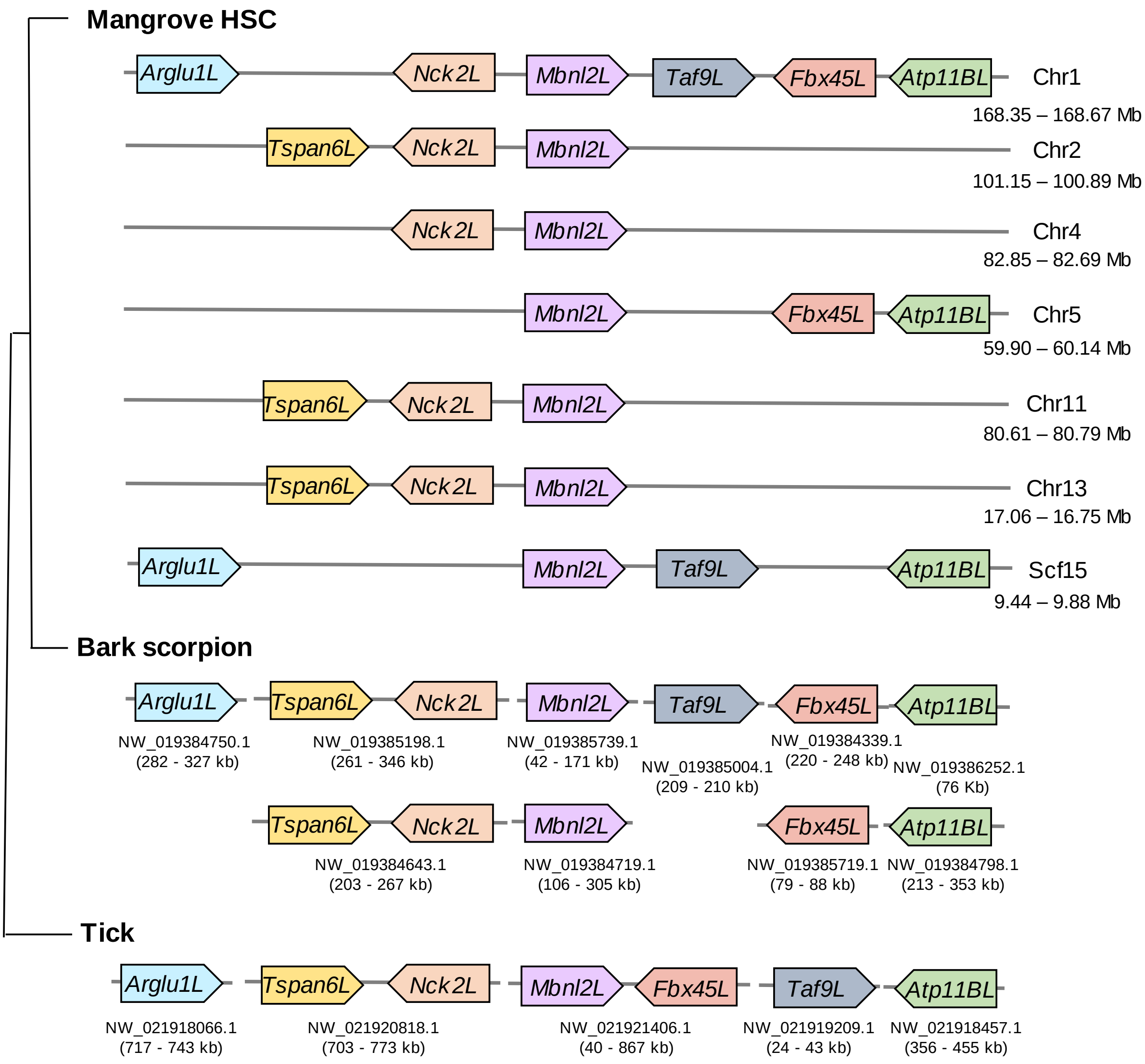

Supplementary Figure 8. Paralogous "*Mbnl2L* loci" in the mangrove HSC genome. *Mbnl2L* gene loci from mangrove HSC, bark scorpion and tick are shown. Block arrows denote genes with their directions representing the transcriptional orientation. Paralogous genes are colour-coded. NCBI accession numbers of bark scorpion and tick contigs or scaffolds are shown below the genes. Since some bark scorpion and tick genes are present on separate contigs or scaffolds, their orientations are presumed. *Arglu1L*, Arginine and glutamate-rich protein 1-like; *Tspan6L*, Tetraspanin 6-like; *Nck2L*, Cytoplasmic protein NCK2-like; *Mbnl2L*, Muscleblind-like protein 2-like; *Taf9L*, Transcription initiation factor TFIID subunit 9B-like; *Fbx45L*, F-box/SPRY domain-containing protein 1-like; *Atp11BL*, Phospholipid transporting ATPase IF-like.

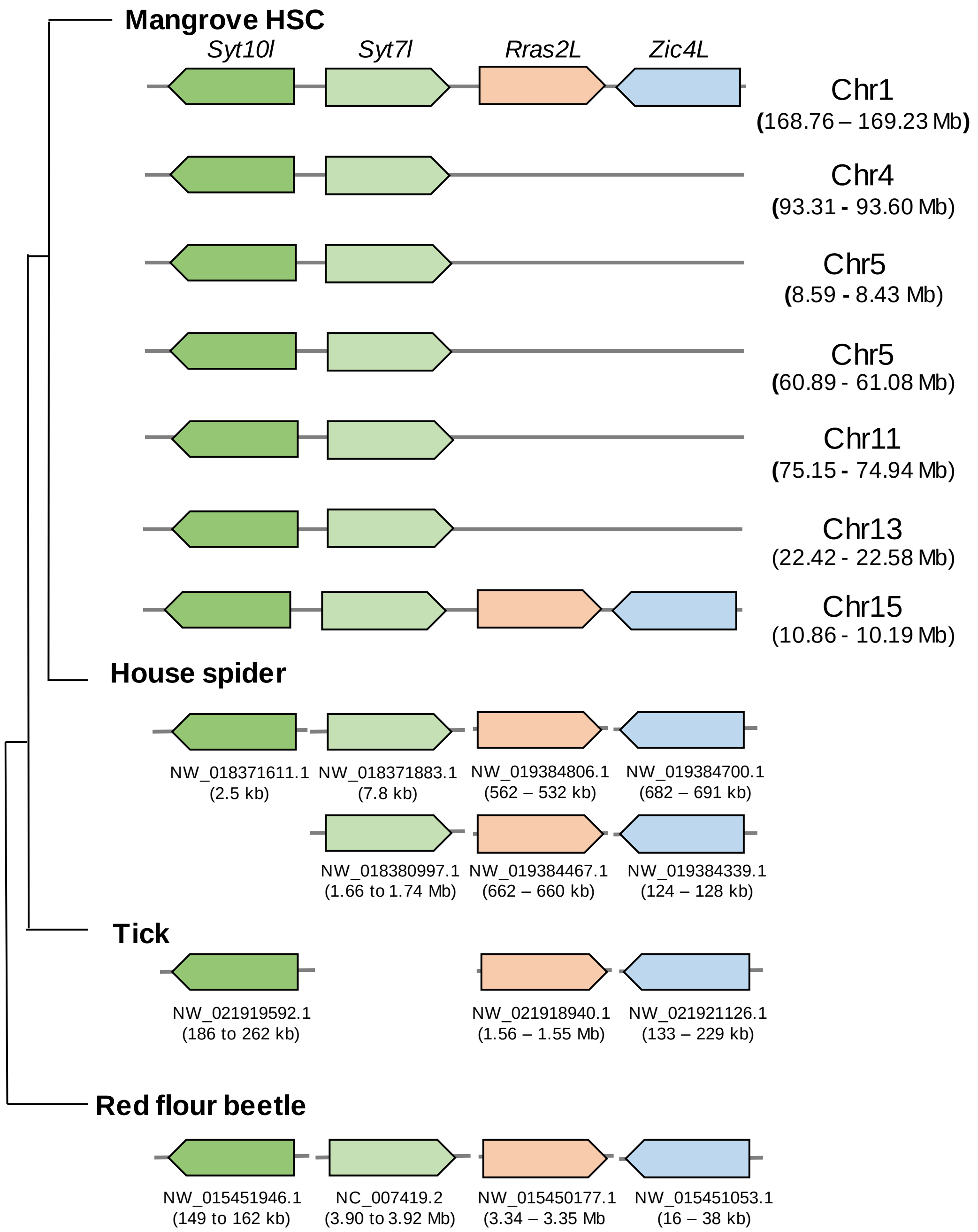

Supplementary Figure 9. Paralogous "*Syt10l*/*Syt7l* loci" in the mangrove HSC genome. *Syt10l*/*Syt7l* gene loci from mangrove HSC, house spider, tick and red flour beetle are shown. NCBI accession numbers of house spider, tick and red flour beetle contigs or scaffolds are shown below the genes. Since the house spider, tick and red flour beetle genes are present on separate contigs or scaffolds, their orientations are presumed. *Syt7l*, Synaptotagmin 7-like; *Syt10l*, Synaptotagmin 10-like; *Rras2L*, Ras-like protein 2-like; *Zic4L*, zinc finger protein zic4-like. The house spider genes *Syt10l* and *Syt7l* genes in the first row are partial.

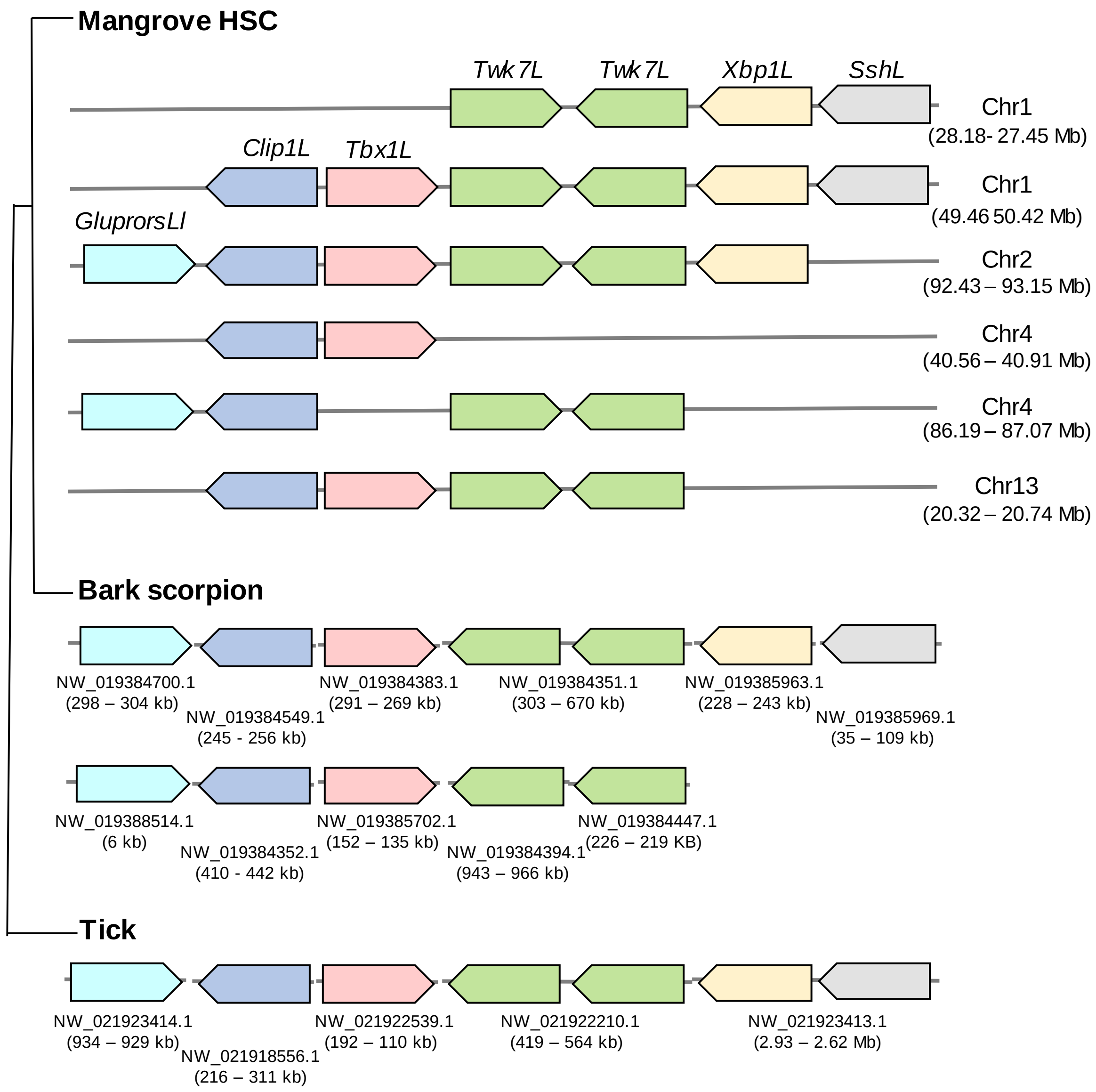

Supplementary Figure 10. Paralogous "*Tbx1L* loci" in the mangrove HSC genome. *Tbx1L* gene loci from mangrove HSC, bark scorpion and tick are shown. Block arrows denote genes with their directions representing the transcriptional orientation. Paralogous genes are colour-coded. NCBI accession numbers of bark scorpion and tick contigs or scaffolds are shown below the genes. Since some bark scorpion and tick genes are present on separate contigs or scaffolds, their orientations are presumed. *Twk7L*, TWiK family of potassium channels protein 7-like; *Xbp1L*, X-box-binding protein 1-like; *Sshl*, protein phosphatase Slingshot-like; *Clip1L*, CAP-Gly domain-containing linker protein 1-lik; *GluprorsL*, bifunctional glutamate/proline--tRNA ligase-like; *Tbx1L*, T-box transcription factor 1.

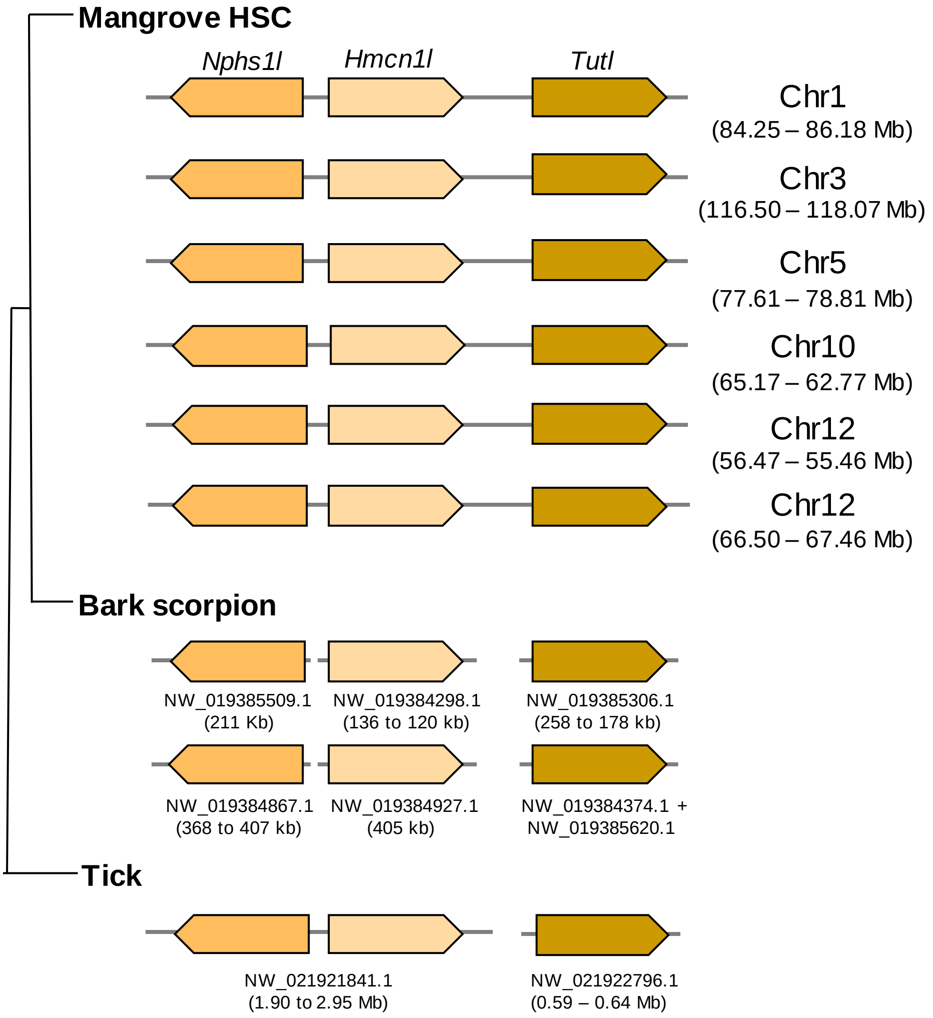

Supplementary Figure 11. Paralogous "*Nphs1l*/*Hmcn1l* loci" in the mangrove HSC genome. *Nphs1l*/*Hmcn1l* gene loci from mangrove HSC, bark scorpion and tick are shown. NCBI accession numbers of bark scorpion and tick contigs or scaffolds are shown below the genes. Since some bark scorpion and tick genes are present on separate contigs or scaffolds, their orientations are presumed. *Nphs1l*, nephrosin-like; *Hmcn1l*, hemocentin1-like; *Tutl*, protein turtle-like.

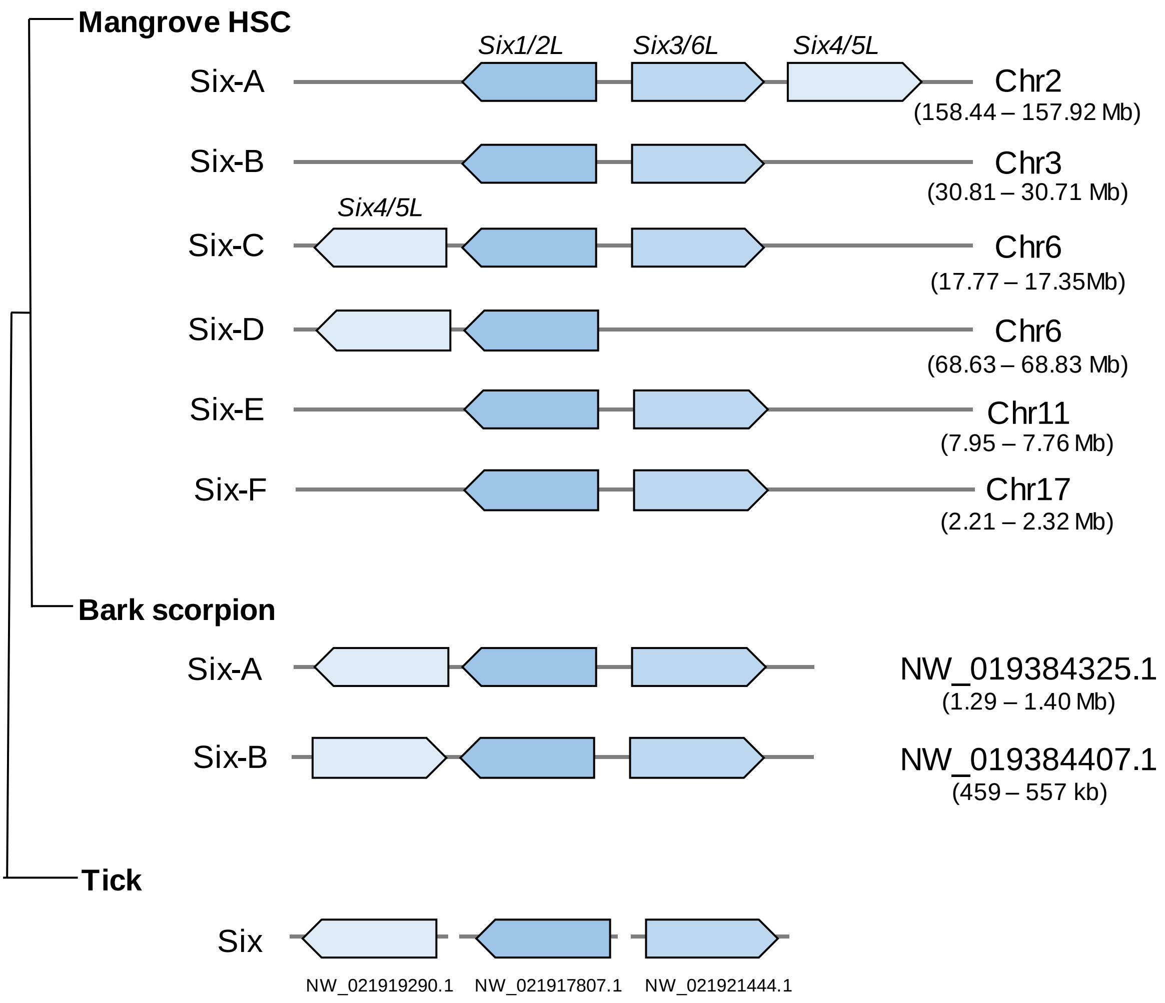

Supplementary Figure 12. Paralogous "Six gene loci" in the mangrove HSC genome. Six gene loci from the mangrove HSC, bark scorpion and tick are shown. NCBI accession numbers are shown for the bark scorpion and tick gene loci. Note that the tick genes are on separate scaffolds. Their orientations shown are presumed.

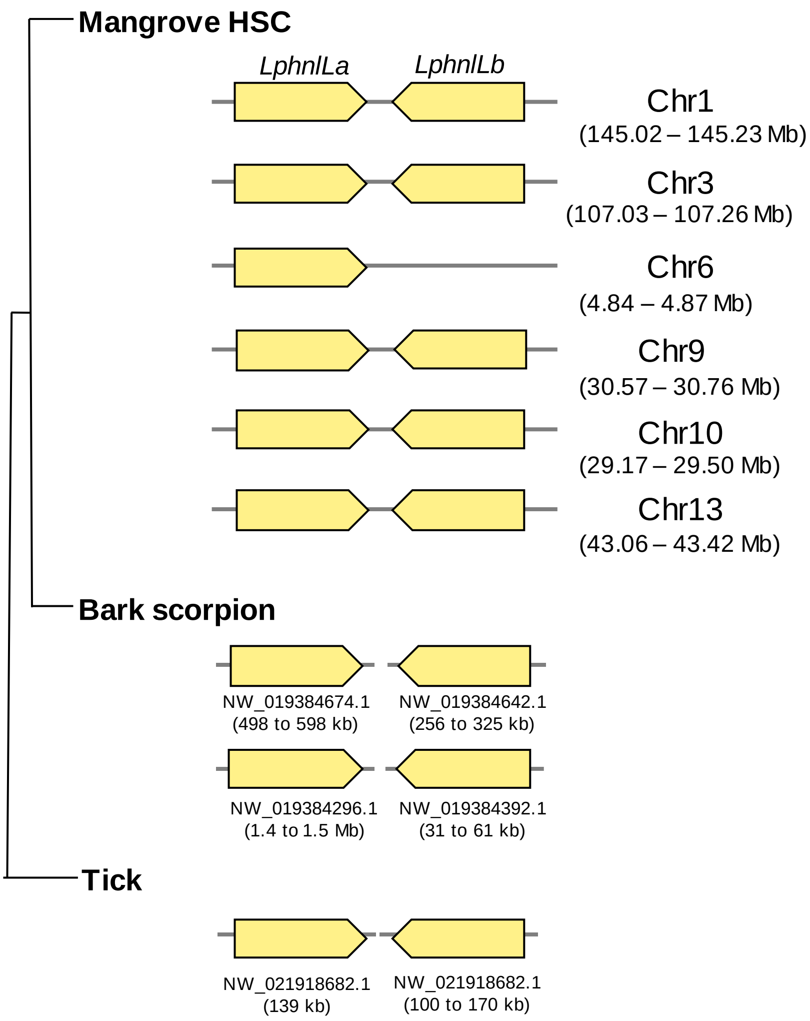

Supplementary Figure 13. Paralogous "*Lphn1La*/*Lphn1Lb* loci" in the mangrove HSC genome. *Lphn1La*/*Lphn1Lb* gene loci from the mangrove HSC, bark scorpion and tick are shown. NCBI accession numbers are shown for the bark scorpion and tick gene loci. Note that each of the bark scorpion and tick genes are on separate scaffolds. Their orientations shown are presumed.

Supplementary Tables

Supplementary Table 1. Genome size estimation using the k-mer method. k-mer value used and the estimated genome size of the mangrove HSC

k-mer (k)	Peak (P)	Read Depth (RD)	Estimated genome size (E) in Gb	First minima	% error	Corrected genome size (E) in Gb
31	92	115.80	1.95	15	2.67	1.90

Supplementary Table 2. Assembly statistics of the PacBio-HiC assembly of the mangrove HSC genome.

Assembled genome size	1.67 Gb
Number of contigs	2,001
Contig N50 length	7.83 Mb
Longest contig length	50.23 Mb
Number of scaffolds	728
Scaffold N50 length	102.34 Mb
Scaffold L90 length	57.5 Mb
Longest scaffold length	188.21 Mb
Total gap length in the assembly	919.24 kb

Supplementary Table 3. Lengths of the 16 assembled mangrove HSC chromosomes

**Chromosome ID**	**Length (Mb)**
Chromosome1	188.22
Chromosome2	175.08
Chromosome3	156.74
Chromosome4	134.27
Chromosome5	105.62
Chromosome6	102.34
Chromosome7	94.78
Chromosome8	94.77
Chromosome9	94.47
Chromosome10	90.88
Chromosome11	86.57
Chromosome12	73.67
Chromosome13	68.32
Chromosome14	57.47
Chromosome15	53.75
Chromosome16	49.92
**Total length**	**1,626.86**
**Chromosome N50 length**	**102.34**

Supplementary Table 4. Comparison of our mangrove HSC genome statistics with other published chelicerate genomes

	Mangrove horseshoe crab (*Carcinoscorpius rotundicauda*) [present study]	Tri-spine horseshoe crab (*Tachypleus tridentatus*)[2]	Tri-spine horseshoe crab (*Tachypleus tridentatus*)[3]	Atlantic horseshoe crab (*Limulus polyphemus*)[4]	Atlantic horseshoe crab (*Limulus polyphemus*)[5]	Mangrove horseshoe crab (*Carcinoscorpius rotundicauda*)[6]	Common house spider (*Parasteatoda tepidariorum*)[7]	Bark scorpion (*Centruroides sculpturatus*)[7]	Tick (*Ixodes scapularis*)[8]	Honeybee mite (*Varroa destructor*)[9]
Assembly size (Mb)	1,668.68	2,167.47	1,942.93	1,828.27	1,229.28	1,577.57	1,445.39	925.47	2081.32	368.94
Number of contigs	2,001	7,523	736,826	455,091	6,614,434	2,317,379	263,908	35,303	6,476	4,498
Total size of contigs (Mb)	1667.76	2,163.81	1,912.88	1,705.99	1,228.82	1,577.57	1,178.90	863.13	2081.32	368.67
Longest contig (Mb)	50.23	16.14	1.16	0.13	NA	0.09	0.02	0.49	12.74	0.93
N50 contig length (Mb)	7.82	1.69	0.05	0.01	0.0004	0.001	0.01	0.05	0.84	0.20
Number of scaffolds	728	204	671,877	286,793	896,522	NA	16,533	8,338	NA	1,426
Longest scaffold (Mb)	188.21	275.86	18.23	5.19	NA	NA	27.00	2.58	NA	76.90
N50 scaffold length (Mb)	102.34	169.00	2.76	0.25	0.003	NA	4.06	0.54	NA	58.54
N50 scaffold number	6	6	190	1,712	NA	NA	94	523	NA	3
N90 scaffold length (Mb)	57.47	101.32	0.003	0.009	NA	NA	0.486	0.133	NA	32.55
N90 scaffold number	14	12	12,426	13,612	NA	NA	448	1,751	NA	7

Supplementary Table 5. Repetitive sequences in the mangrove HSC genome assembly.

**Repeat class**	**Repeat subclass**	**Number of elements**	**Length occupied (bp)**	**Percentage of sequence**
SINEs		409,845	82,646,878	4.95%
	MIRs	2,092	130,208	0.01%
LINEs		267,258	133,810,776	8.02%
	LINE1	1,361	126,034	0.01%
	LINE2	5,104	1,175,672	0.07%
	L3/CR1	38,044	18,969,569	1.14%
LTR elements		423,399	79,602,842	4.77%
	ERVL	81	5,419	0.00%
	ERVL-MaLRs	6	403	0.00%
	ERV_classI	850	63,079	0.00%
	ERV_classII	363	36,411	0.00%
DNA elements		413,861	136,460,698	8.18%
	hAT-Charlie	18,682	12,040,273	0.72%
	TcMar-Tigger	5,758	3,607,403	0.22%
Unclassified		487,595	75,793,881	4.56%
Small RNA		2,937	278,729	0.02%
Satellites		15,899	1,952,409	0.12%
Simple repeats		211,977	8,591,589	0.52%
Low complexity		45,542	2,110,858	0.13%
**Total bases masked**			**520,483,845**	**31.19%**

Supplementary Table 6. Major Pfam domain families identified in mangrove HSC proteins

S. No.	Pfam ID	Pfam Description	Count
1	PF00096	Zinc finger, C2H2 type	2210
2	PF07679	Immunoglobulin I-set domain	858
3	PF14291	Domain of unknown function (DUF4371)	683
4	PF00041	Fibronectin type III domain	674
5	PF00400	WD domain, G-beta repeat	653
6	PF13927	Immunoglobulin domain	633
7	PF00028	Cadherin domain	551
8	PF00069	Protein kinase domain	465
9	PF05699	hAT family C-terminal dimerisation region	423
10	PF00008	EGF-like domain	366
11	PF12796	Ankyrin repeats (3 copies)	363
12	PF01607	Chitin binding Peritrophin-A domain	347
13	PF13855	Leucine rich repeat	343
14	PF00076	RNA recognition motif. (a.k.a. RRM, RBD, or RNP domain)	292
15	PF00435	Spectrin repeat	289
16	PF00057	Low-density lipoprotein receptor domain class A	286
17	PF00001	7 transmembrane receptor (rhodopsin family)	277
18	PF00084	Sushi repeat (SCR repeat)	275
19	PF00046	Homeobox domain	270
20	PF01391	Collagen triple helix repeat (20 copies)	249

Supplementary Table 7. Positively enriched GO terms associated with proteins corresponding to genes in tandem gene clusters

Sr No	GO ID	GO Name	GO category	FDR	P-Value
1	GO:0004499	N,N-dimethylaniline monooxygenase activity	Molecular Function	5.72E-29	1.48E-32
2	GO:0016709	oxidoreductase activity, acting on paired donors, with incorporation or reduction of molecular oxygen, NAD(P)H as one donor, and incorporation of one atom of oxygen	Molecular Function	3.41E-26	1.77E-29
3	GO:0050661	NADP binding	Molecular Function	4.20E-25	3.27E-28
4	GO:0004497	monooxygenase activity	Molecular Function	1.40E-22	1.45E-25
5	GO:0016705	oxidoreductase activity, acting on paired donors, with incorporation or reduction of molecular oxygen	Molecular Function	2.88E-17	3.74E-20
6	GO:0050660	flavin adenine dinucleotide binding	Molecular Function	3.45E-17	5.37E-20
7	GO:0008417	fucosyltransferase activity	Molecular Function	1.11E-16	2.01E-19
8	GO:0043413	macromolecule glycosylation	Biological Process	6.59E-12	1.88E-14
9	GO:0006486	protein glycosylation	Biological Process	6.59E-12	1.88E-14
10	GO:0070085	glycosylation	Biological Process	6.59E-12	1.88E-14
11	GO:0009101	glycoprotein biosynthetic process	Biological Process	6.59E-12	1.88E-14
12	GO:0009100	glycoprotein metabolic process	Biological Process	8.17E-12	2.54E-14
13	GO:0016491	oxidoreductase activity	Molecular Function	3.61E-10	1.22E-12
14	GO:0016758	transferase activity, transferring hexosyl groups	Molecular Function	2.24E-09	8.13E-12
15	GO:0050662	coenzyme binding	Molecular Function	3.26E-09	1.27E-11
16	GO:0055114	oxidation-reduction process	Biological Process	4.82E-09	2.00E-11
17	GO:0016757	transferase activity, transferring glycosyl groups	Molecular Function	1.18E-07	5.21E-10
18	GO:0048037	cofactor binding	Molecular Function	4.26E-07	1.99E-09
19	GO:1901137	carbohydrate derivative biosynthetic process	Biological Process	1.18E-06	5.83E-09

Sr No	GO ID	GO Name	GO category	FDR	P-Value
20	GO:1901135	carbohydrate derivative metabolic process	Biological Process	1.29E-06	6.69E-09
21	GO:0003824	catalytic activity	Molecular Function	2.38E-04	1.36E-06
22	GO:0005184	neuropeptide hormone activity	Molecular Function	6.33E-04	3.78E-06
23	GO:0042303	molting cycle	Biological Process	8.75E-04	6.36E-06
24	GO:0022404	molting cycle process	Biological Process	8.75E-04	6.36E-06
25	GO:0007591	molting cycle, chitin-based cuticle	Biological Process	8.75E-04	6.36E-06
26	GO:0018990	ecdysis, chitin-based cuticle	Biological Process	8.75E-04	6.36E-06
27	GO:0008255	ecdysis-triggering hormone activity	Molecular Function	8.75E-04	6.36E-06
28	GO:0008146	sulfotransferase activity	Molecular Function	0.00218	1.64E-05
29	GO:0007218	neuropeptide signaling pathway	Biological Process	0.00239	1.86E-05
30	GO:0016782	transferase activity, transferring sulfur-containing groups	Molecular Function	0.00287	2.31E-05
31	GO:0005179	hormone activity	Molecular Function	0.00407	3.38E-05
32	GO:0005509	calcium ion binding	Molecular Function	0.00491	4.21E-05
33	GO:0016740	transferase activity	Molecular Function	0.00774	6.82E-05
34	GO:0098742	cell-cell adhesion via plasma-membrane adhesion molecules	Biological Process	0.03462	3.59E-04
35	GO:0007156	homophilic cell adhesion via plasma membrane adhesion molecules	Biological Process	0.03462	3.59E-04
36	GO:0098609	cell-cell adhesion	Biological Process	0.03462	3.59E-04
37	GO:0006030	chitin metabolic process	Biological Process	0.04916	6.01E-04

Supplementary Table 8. Details of the arthropod species used for estimation of the neutral mutation rate and sources of the datasets.

**Arthropod group**	**Species**	**Common name**	**Source**
**Chelicerates**			
Xiphosura, Merostomata	*Carcinoscorpius rotundicauda*	Mangrove horseshoe crab	Present study
Xiphosura, Merostomata	*Limulus polyphemus*	Atlantic horseshoe crab	ftp://ftp.ncbi.nlm.nih.gov/genomes/
Araneae, Arachnida	*Parasteatoda tepidariorum*	Common house spider	ftp://ftp.ncbi.nlm.nih.gov/genomes/
Araneae, Arachnida	*Stegodyphus mimosarum*	Velvet spider	ftp://ftp.ncbi.nlm.nih.gov/genomes/
Araneae, Arachnida	*Acanthoscurria geniculata*	Brazilian white-knee tarantula	https://media.nature.com/original/nature-assets/ncomms/2014/140506/ncomms4765/extref/
Scorpiones, Arachnida	*Centruroides sculpturatus*	Bark scorpion	ftp://ftp.hgsc.bcm.edu/I5K-pilot/Bark_scorpion/
Ixodida, Arachnida	*Ixodes scapularis*	Black-legged tick	ftp://ftp.ncbi.nlm.nih.gov/genomes/
Trombidiformes, Arachnida	*Tetranychus urticae*	Two-spotted spider mite	ftp://ftp.ncbi.nlm.nih.gov/genomes/
**Outgroup**			
Chilopoda, Myriapoda	*Strigamia maritima*	European centipede	ftp://ftp.ensemblgenomes.org/pub/metazoa/release-37/fasta/

Supplementary References

1. Ballesteros JA, Sharma PP. A Critical Appraisal of the Placement of Xiphosura (Chelicerata) with Account of Known Sources of Phylogenetic Error. *Syst Biol*, (2019).
2. Gong L*, et al.* Chromosomal level reference genome of Tachypleus tridentatus provides insights into evolution and adaptation of horseshoe crabs. *Mol Ecol Resour* **19**, 744-756 (2019).
3. Liao YY*, et al.* Draft genomic and transcriptome resources for marine chelicerate Tachypleus tridentatus. *Sci Data* **6**, 190029 (2019).
4. Battelle BA*, et al.* Opsin Repertoire and Expression Patterns in Horseshoe Crabs: Evidence from the Genome of Limulus polyphemus (Arthropoda: Chelicerata). *Genome biology and evolution* **8**, 1571-1589 (2016).
5. Nossa CW*, et al.* Joint assembly and genetic mapping of the Atlantic horseshoe crab genome reveals ancient whole genome duplication. *GigaScience* **3**, 9 (2014).
6. Kenny NJ*, et al.* Ancestral whole-genome duplication in the marine chelicerate horseshoe crabs. *Heredity* **116**, 190-199 (2016).
7. Schwager EE*, et al.* The house spider genome reveals an ancient whole-genome duplication during arachnid evolution. *BMC biology* **15**, 62 (2017).
8. Miller JR*, et al.* A draft genome sequence for the Ixodes scapularis cell line, ISE6. *F1000Res* **7**, 297 (2018).
9. Techer MA*, et al.* Divergent evolutionary trajectories following speciation in two ectoparasitic honey bee mites. *Commun Biol* **2**, 357 (2019).
