# Supplementary material for: Chromosome-level assembly of the horseshoe crab genome provides insights into its genome evolution
Source: Nat Commun. 2020 May 8;11:2322. doi: 10.1038/s41467-020-16180-1 (PMC7210998; doi:10.1038/s41467-020-16180-1)
Supplement: Supplementary file 3 — Reporting Summary [file 41467_2020_16180_MOESM3_ESM.pdf]

nature research

Corresponding author(s): Byrappa Venkatesh

Last updated by author(s): Mar 27, 2020

Reporting Summary

Nature Research wishes to improve the reproducibility of the work that we publish. This form provides structure for consistency and transparency in reporting. For further information on Nature Research policies, see Authors & Referees and the Editorial Policy Checklist .

Statistics

For all statistical analyses, confirm that the following items are present in the figure legend, table legend, main text, or Methods section.

n/a	Confirmed	
x		The exact sample size (*n*) for each experimental group/condition, given as a discrete number and unit of measurement
x		A statement on whether measurements were taken from distinct samples or whether the same sample was measured repeatedly
	x	The statistical test(s) used AND whether they are one- or two-sided<br>*Only common tests should be described solely by name; describe more complex techniques in the Methods section.*
x		A description of all covariates tested
	x	A description of any assumptions or corrections, such as tests of normality and adjustment for multiple comparisons
	x	A full description of the statistical parameters including central tendency (e.g. means) or other basic estimates (e.g. regression coefficient) AND variation (e.g. standard deviation) or associated estimates of uncertainty (e.g. confidence intervals)
	x	For null hypothesis testing, the test statistic (e.g. *F*, *t*, *r*) with confidence intervals, effect sizes, degrees of freedom and *P* value noted<br>*Give P values as exact values whenever suitable.*
x		For Bayesian analysis, information on the choice of priors and Markov chain Monte Carlo settings
x		For hierarchical and complex designs, identification of the appropriate level for tests and full reporting of outcomes
x		Estimates of effect sizes (e.g. Cohen's *d*, Pearson's *r*), indicating how they were calculated

Our web collection on statistics for biologists contains articles on many of the points above.

Software and code

Policy information about availability of computer code

Data collection	No software was used for data collection
Data analysis	"FALCON-Unzip": to generate contig-level genome assembly<br>Redundancy removal pipeline (custom code): to remove redundant contigs<br>SALSA v1, 3D-DNA v180922, Juicebox v1.11.08: to scaffold contigs using Hi-C reads<br>Trinity version 2.2.0: de novo assembly of transcripts<br>RepeatModeler version 1.0.10: prediction of de novo repeats in a genome assembly<br>MAKER v2.31.9: genome annotation<br>InParanoid v4.1: identification of orthologues<br>i-ADHoRe v3.0.01: to identify genomic colinearity and synteny<br>Circos v0.69: Visualization of syntenic relationships<br>4DTv pipeline: to estimate transversion rate at 4D sites extracted from paralogous pairs<br>Tandem gene cluster pipeline (custom code): genome-wide identification of tandem gene clusters

For manuscripts utilizing custom algorithms or software that are central to the research but not yet described in published literature, software must be made available to editors/reviewers. We strongly encourage code deposition in a community repository (e.g. GitHub). See the Nature Research guidelines for submitting code & software for further information.

Data

Policy information about availability of data

All manuscripts must include a data availability statement. This statement should provide the following information, where applicable:

- Accession codes, unique identifiers, or web links for publicly available datasets
- A list of figures that have associated raw data
- A description of any restrictions on data availability

The whole-genome sequence of the mangrove horseshoe crab has been deposited in the DDBJ/EMBL/GenBank database under the accession number VWRL00000000. RNA-seq reads for the four tissues of the mangrove horseshoe crab have been deposited in the NCBI Sequence Read Archive under accession number SRP139459.

Field-specific reporting

Please select the one below that is the best fit for your research. If you are not sure, read the appropriate sections before making your selection.

☐ Life sciences ☐ Behavioural & social sciences ☒ Ecological, evolutionary & environmental sciences

For a reference copy of the document with all sections, see nature.com/documents/nr-reporting-summary-flat.pdf

Ecological, evolutionary & environmental sciences study design

All studies must disclose on these points even when the disclosure is negative.

Study description	High molecular weight DNA as well as RNA were extracted from a single individual which was used for sequencing the genome and transcriptome.
Research sample	Mangrove horseshoe crab (Carcinoscorpius rotundicauda)<br>Phylum: Arthropoda; Sub-phylum: Chelicerata; Order: Xiphosura<br>Stage: Adult<br>Sex: Female
Sampling strategy	A single individual was used for DNA/RNA extraction.
Data collection	N.A.
Timing and spatial scale	Collected in Singapore, 2017.
Data exclusions	No data were excluded.
Reproducibility	N.A.
Randomization	N.A.
Blinding	N.A.

Did the study involve field work? ☐ Yes ☒ No

Reporting for specific materials, systems and methods

We require information from authors about some types of materials, experimental systems and methods used in many studies. Here, indicate whether each material, system or method listed is relevant to your study. If you are not sure if a list item applies to your research, read the appropriate section before selecting a response.

Materials & experimental systems

n/a	Involved in the study	
☒	☐	Antibodies
☒	☐	Eukaryotic cell lines
☒	☐	Palaeontology
☐	☒	Animals and other organisms
☒	☐	Human research participants
☒	☐	Clinical data

Methods

n/a	Involved in the study	
☒	☐	ChIP-seq
☒	☐	Flow cytometry
☒	☐	MRI-based neuroimaging

Animals and other organisms

Policy information about studies involving animals; ARRIVE guidelines recommended for reporting animal research

Laboratory animals	*For laboratory animals, report species, strain, sex and age OR state that the study did not involve laboratory animals.*
Wild animals	The individual was collected from an aquaculture facility at Republic Polytechnic, Singapore. The adult specimen was sacrificed by rapid freezing at -80 degrees Celsius for collecting tissues which were used for DNA/RNA extraction.
Field-collected samples	*For laboratory work with field-collected samples, describe all relevant parameters such as housing, maintenance, temperature, photoperiod and end-of-experiment protocol OR state that the study did not involve samples collected from the field.*
Ethics oversight	No ethical approval was obtained as it is not required for working on an invertebrate in Singapore.

Note that full information on the approval of the study protocol must also be provided in the manuscript.
