# Supplementary material for: Chromosome-level assembly of the horseshoe crab genome provides insights into its genome evolution
Source: Nat Commun. 2020 May 8;11:2322. doi: 10.1038/s41467-020-16180-1 (PMC7210998; doi:10.1038/s41467-020-16180-1)
Supplement: Supplementary file 4 — Description of Additional Supplementary Information [file 41467_2020_16180_MOESM4_ESM.pdf]

Description of Additional Supplementary Files

File Name: Supplementary Data 1
Description: Tandem gene clusters in the mangrove HSC genome.
